# Historical measures of social context in life course studies: retrospective linkage of addresses to decennial censuses

**DOI:** 10.1186/1476-072X-3-27

**Published:** 2004-11-17

**Authors:** Kathryn M Rose, Joy L Wood, Sarah Knowles, Ricardo A Pollitt, Eric A Whitsel, Ana V Diez Roux, DongKeun Yoon, Gerardo Heiss

**Affiliations:** 1 Department of Epidemiology, School of Public Health, The University of North Carolina at Chapel Hill, USA; 2 Department of Medicine, The University of North Carolina at Chapel Hill, USA; 3 The University of Michigan at Ann Arbor School of Public Heath, Ann Arbor, MI, USA; 4 Department of City and Regional Planning, Cornell University, Ithaca NY, USA

## Abstract

**Background:**

There is evidence of a contribution of early life socioeconomic exposures to the risk of chronic diseases in adulthood. However, extant studies investigating the impact of the neighborhood social environment on health tend to characterize only the current social environment. This in part may be due to complexities involved in obtaining and geocoding historical addresses. The Life Course Socioeconomic Status, Social Context, and Cardiovascular Disease Study collected information on childhood (1930–1950) and early adulthood (1960–1980) place of residence from 12,681 black and white middle-aged and older men and women from four U.S. communities to link participants with census-based socioeconomic indicators over the life course.

**Results:**

Most (99%) participants were linked to 1930–50 county level socioeconomic census data (the smallest level of aggregation universally available during this time period) corresponding to childhood place of residence. Linkage did not vary by race, gender, birth cohort, or level of educational attainment. A commercial geocoding vendor processed participants' self-reported street addresses for ages 30, 40, and 50. For 1970 and 1980 censuses, spatial coordinates were overlaid onto shape files containing census tract boundaries; for 1960 no shape files existed and comparability files were used. Several methods were tested for accuracy and to increase linkage. Successful linkage to historical census tracts varied by census (66% for 1960, 76% for 1970, 85% for 1980). This compares to linkage rates of 94% for current addresses provided by participants over the course of the ARIC examinations.

**Conclusion:**

There are complexities and limitations in characterizing the past social context. However, our results suggest that it is feasible to characterize the earlier social environment with known levels of measurement error and that such an approach should be considered in future studies.

## Background

Consideration of the impact of neighborhood social environment on health is now common in social epidemiologic studies [1-7]. While studies of the influence of individual measures of socioeconomic status (SES) on health often include queries for various points during the life course [8-10], estimates of the impact of the neighborhood environment have tended to characterize only the current social context. Current addresses are typically sent to a commercial geocoding vendor and proprietary software is used in conjunction with the Topologically Integrated Geographic Encoding and Referencing (TIGER/Line^®^) files to link the addresses with spatial coordinates within statistical tabulation areas [block group, tract, zip code tabulation area, county]. Notwithstanding concerns about the accuracy in the assignment of statistical tabulation areas by commercial geocoders [11-14], efforts to geocode current addresses are generally successful with reported match rates of 90% or higher at the tract and block group level [1,15].

Obtaining and geocoding historical addresses is more complex and has rarely been undertaken despite the potential advantages derived from its inclusion in life course studies. The completeness and accuracy of historical addresses may not be as high as that of current addresses, unless added care is taken during data collection. Further, widespread use of geocoding in research applications is relatively new and commercial geocoding databases are typically optimized to current street atlases and most recent census tract boundaries. Accurate past addresses, even when assigned correct spatial coordinates, would not be linked with correct historical social contextual data if census tracts had not been defined or summary census data was not available for the area when an individual resided at the address or if the census boundaries had changed over time.

The Life Course SES, Social Context, and Cardiovascular Disease (LC-SES) Study retrospectively collected place of residence during childhood and earlier adulthood on a cohort of middle-aged and older persons. We report on the methods used and our success rate in placing participants into historical census areas and linking them with measures of the social context over time based on self-reported place of residence during childhood and at ages 30, 40, and 50 years.

## Results

The procedures used to obtain the results described in this section are explained in detail in the methods section of this paper as well as in the LC-SES Study manual of procedures, available on the study website [16].

### Linkage of childhood residence to county level census data from 1930–1950

Of 12,681 participants, we excluded 304 who reported living outside of the United States during most of their childhood. Of the remaining 12,377 participants 86% provided apparently correct information on county and state and 10% provided a county which was misspelled. Spelling errors were corrected using a listing of counties in the U.S. available on a publicly accessible website [17]. The remaining 4% of participants did not provide any information on city or county, transposed city and county information, or provided information on a city but not county. Obvious transposition errors were corrected and in cases where the participant provided a city but not a county we searched the publicly available website [17] to identify the matching county. In instances where a city of the same name was listed in multiple counties (n = 27), we did not assign a county. In all, 12,187 (98.5%) of participants reporting a childhood residence in the U.S. were successfully linked with county level U.S. census data. Linkage did not vary by race, gender, adult educational attainment or birth cohort (data not shown).

### Birth cohort and geographical distribution of participants

Participants ranged in age from 45–64 years at the baseline ARIC examination (1987–89). Given their 20 year age span, the years at which they were aged 30 (and 40 and 50) years ranged over several decades, requiring that those from different birth cohorts be linked to data from different census years (Table 1).

**Table 1:** Number of participants assigned to 1960–1980 censuses, overall and by age decade, the LC-SES study, 2001–2002

**Census Year**	**Age 30** ** N**	**Age 40** ** N**	**Age 50 ** **N**	**Total ** **N**
1960	7085	1115	-	8200
1970	5596	5965	1110	12671
1980	-	1891	1386	3277
Total	12681	8971	2496	24148

At baseline, ARIC participants were recruited based on their stable residence in the four study communities. However, at age 30 participants were residing in all 50 states, at age 40 in 47 states, and at age 50 in 31 states. Nonetheless, as shown in Table 2, for all three ages, most participants were already residing in the study areas (as defined by county and state). By age 50, 91% were residing in the study area and only 5% lived out of state.

**Table 2:** Correspondence of county and state of residence at ages 30, 40, and 50 to that at time of ARIC visit 1 exam, the LC-SES study, 2001–2002

	**Age 30**	**Age 40**	**Age 50**
**County and state of residence at ages 30–50 vs. that at ARIC baseline examination**	**N = 12,681 ** **%**	**N = 8,971 ** **%**	**N = 2,496 ** **%**
Residence in same county and state	72	85	91
Residence in different county but same state	11	6	4
Residence in different county and state	17	9	5

### Linkage of addresses at ages 30, 40, and 50 to 1960–1980 census tract data

Table 3 provides a summary of results for linking with 1990 geocoding maps (stage 1 of the process). We submitted 22,140 (92%) of all historical addresses [address refers to a street name and number (if available) and city and state] provided by participants to the geocoding vendor. Those not submitted included P.O. box addresses, as well as those for whom no street information was provided. Of the addresses submitted to the vendor, 75% were assigned spatial coordinates that placed the addresses within a 1990 census tract. About half of the 5,550 addresses that were not assigned coordinates within a census tract were street names without numbers; the rest were cross-streets and apparently complete addresses.

**Table 3:** Historical addresses queried, geocoding success rates and characteristics of addresses not successfully geocoded, the LC-SES study, 2001–2002. ^1^The address information was assigned to the centroid of a zip code area in which all addresses fell within a single block group or census tract or more than 80% of addresses fell within the same census tract.

	**N**	**%age**
Addresses for ages 30, 40, and 50 queried	24,148	100
Partial or complete street address provided	22,140	92
No address, P.O box, or no street name	2,008	8
Commercial Geocoding Results	22,140	100
Address match (geocoded to 1990 census tract)	16,445	74
Usable centroid match^1^	145	<1
Not matched or geocoded to census tract	5,550	25
Characteristics of addresses not matched or geocoded to census tract	5,550	100
Street with number	1,690	30
Cross-street	923	17
Street name without number	2,937	53

Table 4 summarizes our linkage of addresses using the two step process of first linking to the 1990 geocoding maps to get spatial coordinates, and then using the spatial coordinates to obtain the comparable 1960, 1970, and 1980 census tracts. The proportion of addresses judged to be adequate for commercial geocoding (participant recalled at least a partial street address and a city and state) was modestly lower for 1960 than for later years. The proportion that were successfully geocoded to a 1990 tract, and the proportion that were assigned a tract for the historical census, increased steadily from 1960 to 1980. Most addresses with a 1990 tract assignment were placed into the appropriate historical tract for the 1970 and 1980 censuses. Although 61% of 1960 addresses were successfully geocoded to a 1990 tract, only 26% could be assigned a 1960 tract, largely because much of the U.S. was not assigned census tracts in 1960. Use of tract data from the next available census (1970) increased the yield by 16%.

**Table 4:** Percentages of addresses geocoded to 1990 census and then assigned to a 1960, 1970, and 1980 census tract, the LC-SES study, 2001–2002. ^1^Jackson, MS & Washington Co., MD print files of U.S. Bureau of the Census housing data for 1960 [34]. ^2^Includes some addresses sent to vendor but not assigned a latitude and longitude.

	**% of All Addresses**
**Street addresses for 1960 (N = 8200)**	
Sent to geocoding vendor	89
Vendor assigned latitude and longitude	61
1960 tract assigned using overlay/comparability file	26
1960 Assigned area by overlay (non tract area)^1^	16
1970 tract Assigned (non tract area-1960)	16
1960 tract Assigned manually^2^	8
**Total addresses assigned tract**	66

**Street addresses for 1970 (N = 12671)**	
Sent to geocoding vendor	93
Vendor assigned latitude and longitude	71
1970 tract assigned using overlay	69
1970 tract assigned manually^2^	7
**Total addresses assigned tract**	76

**Street addresses for 1980 (N = 3277)**	
Sent to geocoding vendor	94
Vendor assigned latitude and longitude	81
1970 tract assigned using overlay	80
1970 tract assigned manually^2^	5
**Total addresses assigned tract**	85

Manually assigning tracts to addresses modestly increased the proportions that were successfully assigned a census tract for the censuses corresponding to places of residence at ages 30–50 (increase of 8% for 1960, 7% for 1970, and 5% for 1980). Success rates associated with efforts to manually assign historic tracts to addresses varied considerably across study areas and also according to the reasons for the failure of the automated geocoding procedure. Of the addresses which we attempted to manually assign a census tract, we were successful for 54% of Forsyth, NC addresses, 45% of Jackson, MS addresses, 30% of Minneapolis, MN address and 29% of Washington County, MD addresses (data not shown). Rates were particularly low in MD because many roads were located in areas not classified into tracts in the 1960 census and because the conversion to a grid address system – during which time some streets renumbered and renamed – did not occur until the early 1990s. In contrast, the low success rate in MN occurred primarily because the tracts were physically small and streets tended to cross multiple tracts.

**Figure 1:**
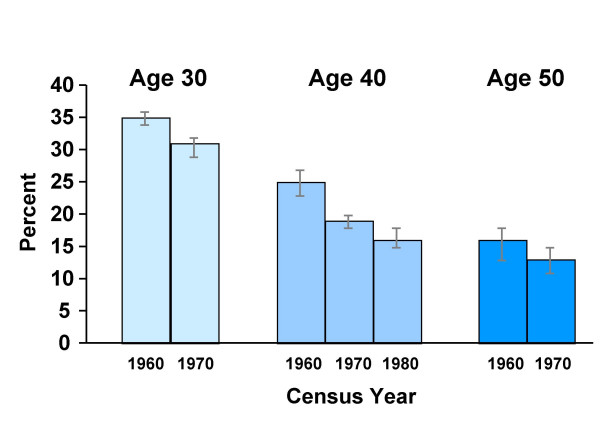
Percentage of addresses not assigned a census tract by age and census year, the LC-SES study, 2001–2002.

Figure 1 shows the proportion of participants not linked to a historical census tract by adult age and census year. Overall, the rate of successful assignment to a historical census tract was lower at younger ages (67% for age 30, 81% for age 40, 86% for age 50) and within each age decade, for earlier censuses.

### Variation in linkage to census tracts by sociodemographic characteristics

Table 5 presents childhood and midlife socio-demographic characteristics of participants by success of census tract assignment at ages 30, 40, and 50. There was no difference in the proportion of participants assigned a census tract by mean age at baseline. African-Americans comprised modestly higher proportions of those groups not assigned to census tracts. For ages 30 and 40, a modest but greater proportion of men were in the group not assigned a census tract. There were differences in both educational attainment and family income between those assigned and not assigned census tracts. Those in the lowest strata of income and education tended to be more heavily represented in the group not assigned census tracts; this pattern was also observed for those in the highest educational group at age 30.

**Table 5:** Comparison of sociodemographic characteristics of those with and without tract assignment^1^, the LC-SES study, 2001–2002. ^1^Chi Square test used to statistically compare differences in proportions and t-test used to statistically compare differences in means of those assigned and not assigned census tracts. ^2^Ns for each characteristic vary slightly due to missing data. * p < 0.05; ** p < 0.01; *** p < 0.001; **** p < 0.0001

	**Age 30**		**Age 40**		**Age 50**	
	**Tract assigned**		**Tract assigned**		**Tract assigned**	
**Characteristics at baseline examination**	**Yes**	**No**	**Yes**	**No**	**Yes**	**No**

Total N (%)^2^	8,448 (67)	4,233 (33)	7,262 (81)	1,709 (19)	2,143 (86)	353 (14)
Mean age	53	54	56.3	57	62	62
% Male	41	46****	43	47*	46	46
% African American	25	26	23	25	21	25
Educational attainment (%)						
< 12 years	21	21****	23	26***	28	35***
12 years or equivalent	44	36	42	37	40	40
> 12 years	35	43	35	37	32	35
Family income (1987–89) (%)						
< $16,000	19	21**	20	25****	28	36*
$16,00–49,999	55	51	54	53	56	51
$50,000	26	28	25	22	16	13
% Born outside of study state	20	38****	23	36****	23	35****
Father's education (%)						
0–9 years	53	48****	55	52	57	61
9–12 years	35	34	32	34	29	23
> 12 years	12	17	13	14	14	15
Father's occupation (%)						
Professional & management	11	14	11	12	10	15
Technical & sales	11	11	11	11	12	8
Mechanical & crafts	21	18	20	19	21	15
Farming	30	31	32	33	34	39
Laborers, operators & drivers	21	19	20	19	17	14
Service	7	7	7	6	7	8
% Parents owning home	92	86****	93	82****	93	80****

There were variations, albeit inconsistent, in early life sociodemographic characteristics and assignment to census tract of residence at ages 30, 40, and 50. Those living outside of their current (at baseline) state of residence during childhood were markedly less likely to be linked with a census tract at ages 30–50, while those whose parents were homeowners were more likely to be assigned tracts. Those who had fathers with twelve or more years of education or who were in managerial and professional or in farming occupations were modestly but more heavily represented in the groups not assigned tracts. In contrast, those with fathers who were in blue collar occupations (mechanical and crafts; laborers, operators, and drivers) were consistently less likely to be in the group not assigned tracts. These differences by fathers' occupations, while consistent, were generally modest.

## Discussion

Assessment of social circumstances in childhood and early adulthood in life course studies is typically limited to individual level measures of parental / own occupation or education [8-10]. The contribution of the contemporaneous social context to a variety of health outcomes [1,4-6] suggests that evaluation of the impact of earlier socio-environmental exposures on health is also of interest. Its inclusion in life course paradigms is not novel [18,19] but its implementation in population-based studies in the U.S. is, in part because of uncharted approaches to the measurement of historical context on a scale suitable to epidemiologic studies. We report on our methods, completion, and error rates in retrospectively collecting former places of residence in a middle-aged and older cohort and linking this information with census data. We successfully linked 99% of participants with 1930–1950 county level census data corresponding to their childhood place of residence. Successful linkage of addresses from ages 30–50 with corresponding census tract level data from the 1960–80 censuses was lower. Approximately two-thirds of participants were assigned a census tract for 1960, 76% for 1970 and 85% for 1980. For purposes of comparison, ARIC participant addresses at the time of each of the ARIC examinations (1987–1999) achieved geocoding match rates (by the same vendor) of 94%.

Match rates of participants' addresses to the 1960–1980 U.S. census tracts were lower largely for three reasons: limited ability to recall complete historical addresses, obsolete or unusable addresses (e.g., change in street numbering, renaming of rural routes), and the previously incomplete coverage of census tracts in the U.S. Linkage rates were considerably higher in 1970 when census tracts were in place at all of our study sites. Now the coverage of census tracts is complete for the U.S. and grid address systems are common even in rural areas. Thus, studies of more recent birth cohorts though still faced with limitations of recall would be expected to have higher linkage rates.

The yield from attempts to commercially geocode incomplete street addresses (e.g., street name but not number) were quite low, even when street fell completely within the boundaries of one census tract. TIGER/Line^® ^files represent streets as a series of segments. When streets consisted of more than one segment, even when all were located within a single tract, it appears that commercial geocoding software was not able to assign a census tract. We were able to assign census tracts to a sizable portion of these addresses by using detailed street maps overlaid with historical census boundaries. However, this process involves multiple steps and is labor intensive and thus can be practically implemented only in areas where a sizable number of addresses are located.

Recall of county, city, and state of residence during childhood was virtually complete, while recall of street address of former places of residence was more limited. The potential limitations of retrospectively recalled data are known [20,21], suggesting the need to assess the accuracy of addresses corresponding to former places of residence information provided by interviewees. Review of a subset of decedents indicated that recall of county and state of birth showed greater than 90% concordance with that recorded on their birth certificate (KM Rose, unpublished data). While it is technically possible to use historical city directories to verify addresses, privacy concerns prevent us from linking addresses to participant names. In future studies, advanced notification to the interviewee should be considered as it would offer them the opportunity to consult records and / or a spouse, potentially reducing the degree to which some individuals may not be able to recall a complete street address.

Linkage to county-level place of childhood residence did not vary by participant sociodemographic characteristics. In contrast, successful linkage of the later but more detailed address information to 1960–1980 census tract data varied by sociodemographic characteristics (gender, family income, father's and own education). More striking was the substantial difference seen between those born in vs. outside the study state. Those born outside of the study states were between 1.5 and 1.9 times more likely to not be assigned a tract than those born in one of the study states. To some extent, this occurred because a higher proportion of the participants born in other states originated from areas lacking census tracts at the time of the pertinent historical census.

The optimal geographical unit of analysis for contextual measures is discussed in the literature [22-24]. Studies tend to use either census tracts or block groups, and reports suggest that the two produce similar results [1,22]. There is concern that data aggregated at the county level is not optimal to characterize the social environment. However, ecological studies as well as those including an assessment of individual-level SES [25-29] have reported inverse associations between county-level socioeconomic characteristics and health outcomes. Since childhood county of residence is recalled quite well per our results and it corresponds to the smallest level of geographical aggregation at which census data is available prior to 1960, its use as a measure of the social environment in life course studies deserves consideration.

Our purpose in assigning current and former adulthood places of residence to census tracts was to link participants with census-based neighborhood profiles to provide area-based measures of the social context (s) across epochs spanning early to later adulthood. Although the approach presented here had not been previously attempted and has logistical complexities, its feasibility, success and error rates are now documented. The opportunity to acquire area-based measures of SES in a historical context does not obviate the methodologic challenges associated with life course research, however. Among the latter it is worth mentioning that many census variables differ across censuses (see table entitled "SES var by census in the Census Tract SES section of the LC-SES Study website [30]). For example, the percentage living below the poverty level was not calculated until the 1970 census and prior to 1940, years of education were not collected. Also, the meanings and distributions of census variables are subject to secular change (i.e., over time the average educational level of the U.S. population has increased, mean/median incomes and housing values change across time). Thus, careful consideration of birth cohort effects and of the social and economic contexts at each point of data collection is required.

## Conclusions

The importance of the social and economic environments in influencing health is increasingly recognized, yet most research to date is limited to the current social context [1-6]. We believe that this deficit is largely driven by the greater complexity and limitations inherent in retrospectively characterizing the past social context. The experience of the LC-SES study suggests that it is feasible to do this effectively. Studies incorporating such an approach offer the potential of improved understanding of socioenvironmental influences over the life course on health, and should be considered.

## Methods

### Study participants

The Atherosclerosis Risk in Communities (ARIC) study is an investigation of the etiology and natural history of atherosclerosis and its sequelae. At baseline (1987–89), 15,792 African American and white middle-aged men and women from four U.S. communities (Forsyth County, NC; Jackson, MS; the northwest suburbs of Minneapolis, MN; and Washington County, MD) were included. An account of the design and procedures is published [31]. Since baseline, the ARIC study telephones participants annually to establish vital status and assess indices of cardiovascular disease, including hospitalizations. Institutional Review Boards (IRB) at each ARIC centre approved the study, and the investigators obtained informed, written consent from all participants.

An ancillary study to ARIC, the LC-SES Study was initiated in Spring 2001 to examine the association between SES across life and adult CVD-related conditions, and to determine the extent to which the current and historical context [neighborhood estimated at the county (early childhood) and census tract (early adulthood) level] modify the association of individual-level SES exposures and CVD. Trained interviewers administered a telephone questionnaire including 44 questions about parental and early adulthood occupational and educational exposures, current sociodemographic characteristics and childhood and earlier adulthood places of residence. Participants responding to the questionnaire (N = 12681), represent 81% of the ARIC baseline cohort and approximately 94% of cohort survivors. Additional details about the LC-SES Study can be found in the manual of procedures [16] and other documents available on the study website [32].

### Ascertainment of childhood and early adulthood residences

Participants were asked "Where did you mostly live when you were a child? If possible, give me the city/town, county, and state of residence." Participants were also asked to provide their address (street number and name, city, county, state, and zip code) at various points during adulthood. Everyone was asked to provide addresses for age 30 (n = 12,681), and those who had first participated in the ARIC study after age 49 (n = 8,971) or 59 (n = 2,496) were also asked to provide addresses for ages 40/50 and 50, respectively. Those unable to provide an exact address were asked to provide the street name and the closest cross-street.

### Editing & linking childhood county of residence with 1930–50 censuses

The year at which participants were aged ten years, which represented the approximate midpoint of childhood, was determined in order to link with the county-level socioeconomic data from the closest census year (1930, 1940, 1950). When a city, but not a county was provided, we used a publicly available website to attempt to identify the correct county [17]. County was chosen, as it was the smallest level of aggregation universally available in published census data before 1960. These data were obtained electronically through the Inter-University Consortium for Political and Social Research (ICPSR) at the University of Michigan.

### Preparing addresses at ages 30, 40, 50 for geocoding

Prior to geocoding, all state data was standardized to conform to the two-digit U.S. Postal Services state coding system. Within each state the accuracy of the spellings of cities were verified. Street addresses were reviewed and computer programs written to correct obvious misspellings and to standardize formats. We did not submit zip codes because those accompanying the historical address could have changed over time. Because our goal was to classify the social environment where participants lived, we excluded post office box addresses as they do not necessarily correspond to actual residences. These along with other incomplete and unusable addresses (e.g., institutions, military APO, c/o, etc.) were not sent for geocoding. After editing, addresses and an encrypted study ID number were sent to a commercial vendor under contractual terms of confidentiality negotiated by university counsel and approved by the IRB.

### Geocoding

The vender assigned to each address: spatial coordinates, Federal Information Processing Standards (FIPS) codes for statistical tabulation areas corresponding to 1990 census boundaries, and a match code describing the degree of accuracy of the geocoding. The accuracy rating assigned by the vendor ranged from" house range address matches" (best) to the "centroid of county" (worst). As we were interested in accurately classifying each participant's place of residence at the level of the census tract (the smallest geographical unit at which data for all censuses since 1960 was available), we accepted only house range address matches (e.g., accuracy at level of exact address, intersection, or street segment) or matches to centroids of zip code areas where everyone lived within a census block group, census tract or where more than 80% of addresses in area were located in the same tract. Rural routes were sent to the vendor but these addresses were not successfully geocoded.

### Comparison of geocoding methods

Two methods were considered to link the spatial coordinates obtained from the vendor with the appropriate historical census tract. The overlay method uses the spatial coordinates assigned to exact address matches in conjunction with historical boundary maps to place addresses into historical tracts. The comparability file method uses current US Bureau of the Census tract assignments that are traced back in time stepwise to 1980 tracts, then from 1980 to 1970 tracts using files that describe tract changes from decade to decade. As a test, we compared the 1970 tract assignments by the two methods for 13,044 addresses that were successfully geocoded to the 1990 census by the geocoding vendor. While all addresses were assigned tracts using the overlay method, 36% could not be assigned a 1970 tract using the comparability files due to census tract merges (a tract contains parts of more then one tract from the previous decade). Of the remaining addresses (n = 8348), 97% were assigned the same tract by both methods. Because there are known errors in the assignment of spatial coordinates by commercial geocoding vendors [11,12,14], we could not rule out minor errors in the placement of tract boundaries included in polygon files. We also found an error in a comparability file during this test. We chose the overlay method to link with 1970 and 1980 censuses because it allowed us to locate addresses that could not be assigned tracts using the comparability files with no obvious lack of accuracy.

### Linking addresses at ages 30, 40 and 50 with 1960–80 census tracts

**Figure 2:**
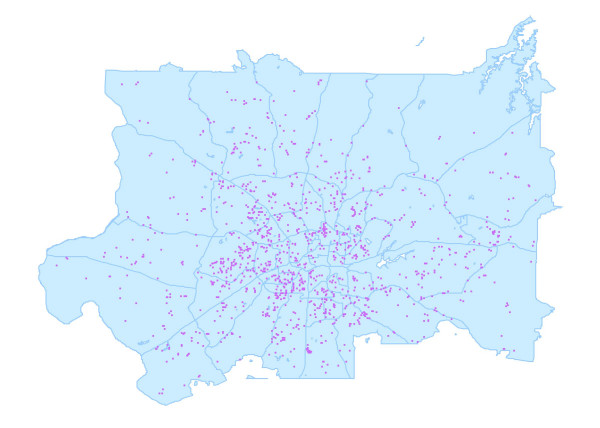
1970 census tracts in Forsyth County, NC and geocoded 1970 participant addresses, the LC-SES study, 2001–2002.

We determined the census year (1960, 1970, 1980) that corresponded most closely to when the participant resided at each address. Arcview GIS Version 3.3 software was used to overlay the spatial coordinates assigned to addresses by the vendor onto Geolytics, Inc. shape files of census tract and block numbering area boundaries of the appropriate census year [Census CD 1970, Census CD 1980]. The spatial coordinates falling within the historical tracts were assigned the corresponding tract number. Figure 2 provides an example of the overlay of the spatial coordinates of Forsyth County, NC addresses that were matched with the 1970 census boundaries.

Electronic shape files were not available for the 1960 census. Thus, 1960 addresses were placed into 1970 tracts using the overlay method and then mapped to the appropriate 1960 tracts using files providing data on the correspondence between 1970 and 1960 tracts. These were obtained from print volumes of comparability files published by the US Bureau of the Census [33] and keyed into a database. If the 1970 tract was a merged tract it was not possible to uniquely identify the 1960 tract. In these circumstances we attempted to manually place the address in a tract as described below.

### Assigning tracts when commercial geocoding efforts failed

When a 1960 address fell into a 1970 tract made up of merged 1960 tracts or when addresses were not geocoded by the commercial vendor [street name but not number, cross streets, obsolete addresses (road renamed or renumbered)], we attempted to manually place addresses into historical census tracts. Because this process is labor intensive, we undertook this effort only for addresses which were located within the four ARIC study communities (as a large number of addresses were not clustered in other areas). First, we obtained detailed street maps for the four study areas and overlaid them with census tract boundaries and numbers from the three historical censuses. Then, using web-based Mapquest^® ^tools and the street map legends, we attempted to locate each address. If a street was contained within the boundary of a census tract, we assigned it the corresponding tract number. If a street crossed a census tract boundary or was the boundary for two or more tracts, we did not assign a census tract.

A large number of historical addresses in Washington County, MD were obsolete, because in the early 1990s the state changed to a grid address system to improve emergency response systems. Thus, we obtained historical street maps from the Hagerstown, MD Public Library and tried to locate the original street names in an attempt to manually assign a census tract using the procedure described above.

### Linking with 1960–1980 socioeconomic census data

For addresses placed within a 1960–1980 census tract, we linked with tract level socioeconomic data. For 1970 and 1980 we used data from Geolytics, Inc. (Census CD 1970, Census CD 1980). The ODUM Institute at the University of North Carolina, USA provided electronic 1960 census tract data. Jackson MS and Washington County MD had not been assigned census tracts in 1960. For Jackson, MS and the portion of Washington County, MD falling within the Hagerstown city limits, we obtained 1960 census housing data at the level of city blocks from print volumes [34], and aggregated them into tract data using the1970 tract boundaries. However, for other areas without census tracts in 1960 this information was either not available (e.g., areas near Hagerstown but outside of the city limit) or it was not feasible to collect it from print volumes because of a small number of participants in the areas. For these addresses, data from the next closest census, 1970, were substituted.

## Authors' contributions

KMR conceived of and led the writing of the manuscript. JLW analyzed the data on early adulthood and developed the methods for assigning census tracts to historic addresses. GH, the principal investigator of the LC-SES Study, contributed to the conceptualization and writing of this manuscript. EAW assessed the accuracy of the commercial geocoding and developed standardized procedures for manual geocoding. SK developed standardized procedures for editing the recalled address data. RP analyzed data pertaining to childhood place of residence. DY was instrumental in reviewing methods for placing participants into their historical tracts. AVDR provided expert input on methods of geocoding. All authors helped to frame the ideas, interpret findings, and review drafts of the manuscript.
